# Synchrony 2022: Epilepsy and Seizures in Autism Spectrum Disorder Roundtable

**DOI:** 10.3390/jpm13030557

**Published:** 2023-03-20

**Authors:** Richard E. Frye, Heer Nanda, Samuel J. Pleasure, Manuel F. Casanova, Richard G. Boles, Jeffrey Lewine, John Gaitanis, James B. Adams

**Affiliations:** 1 Autism Discovery and Treatment Foundation, Phoenix, AZ 85050, USA; 2 Rossignol Medical Center, Phoenix, AZ 85050, USA; 3 Southwest Autism Research and Resource Center, Phoenix, AZ 85016, USA; 4 University of California Berkeley, Berkeley, CA 94720, USA; heer.nanda@berkeley.edu; 5 Department of Neurology, University of California San Francisco, San Francisco, CA 94158, USA; samuel.pleasure@ucsf.edu; 6 Department of Biomedical Sciences, University of South Carolina School of Medicine Greenville, Greenville, SC 29605, USA; manuel.casanova@louisville.edu; 7 NeurAbilities, Vorhees, NJ 08043, USA; rboles@neurabilities.com; 8 NeuroNeeds^®^, Old Lyme, CT 06371, USA; 9 Center for Advanced Diagnostics, Evaluation and Therapeutics, Albuquerque, NM 87501, USA; jlewine@cadetnm.com; 10 Hasbro Children’s Hospital, Brown University, Providence, RI 02903, USA; jgaitanis@lifespan.org; 11 School for Engineering of Matter, Transport & Energy, Arizona State University, Tempe, AZ 85287, USA; jim.adams@asu.edu

The BRAIN Foundation (Pleasanton, CA, USA) hosted Synchrony 2022, a translational medicine conference focused on research into treatments for individuals with neurodevelopmental disorders (NDD), including those with autism spectrum disorders (ASD). One of the four roundtables focusing on some of the most difficult and unsolved problems in ASD focused on advancing the understanding and treatment of epilepsy and seizures in individuals with ASD. This is because epilepsy and seizures in ASD are more severe and difficult to treat as compared to those in typically developing individuals.

Dr. Richard E. Frye, MD, Ph.D., President of the Autism Discovery and Treatment Foundation, chaired a roundtable with experts in epilepsy and ASD treatments. The selected experts gave presentations focusing on important topics (Table 1). However, significant time was also devoted to discussing important knowledge gaps, input from parents regarding unmet needs, and case presentations from physicians and parents to illustrate some of the most difficult cases.

Dr. Samuel Pleasure, MD, Ph.D., Professor of Neurology at the University of California, San Francisco, provided an overview of the role of autoimmunity in epilepsy. This included the specific characteristics of autoimmune epilepsy, specific autoantibodies involved in it, its syndromes, scoring systems used to assist in its diagnosis, and high-throughput techniques used for the discovery of novel autoantibodies that might drive it.

Manuel F. Casanova, MD, Professor of Biomedical Sciences at the University of South Carolina, reviewed studies on ASD that indicated a high prevalence of brain malformations. Differences in cortical thickness, surface area, and cortical folding in neuroimaging studies indicate a disruption of normal brain development. Higher resolution studies using postmortem techniques evidence disturbances of both cell division and migration. A prominent commonality between human and animal models of ASD is the reduction in the number of parvalbumin interneurons. In ASD, the loss of these fast-spiking interneurons offers a neuropathological mechanism common to both seizures and executive dysfunction.

Richard G. Boles, MD, Director of the NeuroGenomics Program at NeurAbilities and Chief Medical and Scientific Officer of NeuroNeeds, provided an overview of both genetic causes and contributors of epilepsy with a specific focus on ASD. He reviewed the large studies that outlined the genetic causes of epilepsy, specific cases of ASD in which novel de novo presumably causal variants were identified after extensive re-analysis of raw whole genome sequencing data, and inherited non-causal disease variants that may have provided a significant contribution to the underlying pathophysiological disease processes. Most significantly, Dr. Boles discussed various treatment approaches that could be implemented and improve outcomes in patients based on their identified genetic variation. He presented specific cases in which dramatic improvements in function and outcome resulted from the implementation of genetically guided treatments.

Jeffrey Lewine, CEO of the Center for Advanced Diagnostics, Evaluation, and Therapeutics, discussed both clinical seizures and subclinical epileptiform activity in his presentation. Dr. Lewine demonstrated the significant advantage of using magnetoencephalography (MEG) for detecting subclinical discharges because of its increased sensitivity over standard and extended encephalography. Dr. Lewine demonstrated how MEG reveals patterns that correspond with clinical characteristics, such as language impairment and neurodevelopmental regression, and are predictive of the response to steroid treatment.

John Gaitanis, Chief of Child Neurology at Hasbro Children’s Hospital, Brown University, discussed several topics. Dr. Gaitanis introduced the novel hypothesis that individuals with ASD, neurodevelopmental regression, epilepsy, and aggressive behavior are linked. Dr. Gaitanis then went on to discuss the history of cannabis use in the treatment of epilepsy, demonstrating that it had originally been utilized to control the disorder in ancient China in 2838 BC by Shen Nung, the “red emperor”. Dr. Gaitanis went on to describe the important active components in cannabis and their mechanism of action on the brain. Dr. Gaitanis reviewed the preclinical and clinical studies supporting the use of cannabis in the treatment of epilepsy, including its marked efficacy for treating Dravet’s syndrome and Lennox–Gastaut syndrome.

James Adams, Director of the Autism Program at Arizona State University, discussed dietary and nutritional changes in children with ASD and epilepsy and the implications of these changes on treatment. Dr. Adams reviewed the evidence for the use of the ketogenic diet and the modified Atkins diet for the treatment of epilepsy. Dr. Adams discussed the importance of key nutrients in pathways important for controlling seizures, including vitamins B_1_, B_9_, E, and D; biotin; magnesium; manganese; selenium; calcium; and carnitine, and posited that a targeted multivitamin/mineral/micronutrient could be a safe and effective adjunctive treatment to other more traditional anti-epileptic drugs in order to improve seizure control in individuals with ASD.

Lastly, Dr. Richard E. Frye discussed the specific metabolic disorders that are commonly associated with ASD and epilepsy, particularly cerebral folate deficiency, mitochondrial disorders, and redox abnormalities, including potential treatment approaches that have the potential to improve the disorder.

The discussion throughout the roundtable session converged on several important points that parents, clinicians, and scientists agreed should gain more attention:

- Better defining the characteristics, etiology, diagnosis, and treatment of epilepsy in ASD, particularly in how they differ from epilepsy in those without ASD;
- Considering whether one unprovoked seizure should indicate initiation of anti-epileptic treatment given the high risk of its recurrence in individuals with ASD;
- Defining the difference between individuals with ASD who have early-onset and late-onset epilepsy;
- Defining the association between epilepsy and behavioral exacerbations;
- Defining the influence of common comorbid conditions on epilepsy, especially with the possibility of leveraging treatment of these comorbid abnormalities to improve treatment of epilepsy in ASD;
- Defining medical care models for improving seizure control in those with ASD.

In an effort to move forward, many of the panelists agreed to work together to develop several review papers outlining current knowledge, defining knowledge gaps, and providing recommendations based on the available data. The ultimate goal of the group will be to develop a guideline that will be useful for clinicians in improving the care of children with ASD and epilepsy.

## Figures and Tables

**Table 1:** Focused presentations at the roundtable session.

Speaker	Topic
Samuel Pleasure, MD, Ph.D.	Autoimmunity in Epilepsy
Manuel Casanova, MD	Neuropathology of Autism and Epilepsy
Richard G. Boles, MD	Genetics of Epilepsy in Autism
Jeffrey Lewine, Ph.D.	Magnetoencephalography
John Gaitanis, MD	Cannabis and Treatment of Epilepsy
James B. Adams, Ph.D.	Nutrition and Vitamins and Epilepsy
Richard E Frye, MD, Ph.D.	Metabolic Abnormalities and Epilepsy

